# Clinical Experience of Using an 8 French Newton Shaped Catheter (Neuro-EBU) During Endovascular Treatment: A Case Series of 21 Patients

**DOI:** 10.7759/cureus.26049

**Published:** 2022-06-17

**Authors:** Kazushi Maeda, Yosuke Kawano, Yoshio Suyama, Satoru Kawarazaki, Kohei Irie, Kazuhisa Kuwabara, Shintaro Nagaoka, Hidefuku Gi, Yukihide Kanemoto

**Affiliations:** 1 Neurological Surgery, Baba Memorial Hospital, Sakai, JPN; 2 Neurosurgery, Baba Memorial Hospital, Sakai, JPN

**Keywords:** neurointervention, endovascular, wire exchange, guide catheter, newton shaped catheter

## Abstract

The first step in endovascular treatment is the stable placement of a guide catheter (GC) into the target parent vessel. However, sufficient GC stabilization is sometimes difficult to obtain when the approach route has severe tortuosity. Here, we report our experience with and the usefulness of the 8 French (Fr) Newton-shaped Neuro-EBU vascular catheter (SILUX Co., Ltd., Kawaguchi City, Saitama Prefecture, Japan), which is rigid over its entire length except near the tip, in a case series of 21 patients. Of the 21 cases, 19 cases were successfully treated using the Neuro-EBU.

The 8 Fr Newton-shaped Neuro-EBU might be useful both as a special GC and as a wire exchange catheter when placement of the conventional GC is difficult due to severe tortuosity of the access route. Although rarely used, the Neuro-EBU catheter can serve as a practical alternative when the access route is challenging. To the best of our knowledge, there are no detailed reports on the technical use of the Newton-shaped vascular catheter in the field of neurointervention. We present the usefulness of the specially shaped 8 Fr guide catheter.

## Introduction

Stable placement of a guide catheter (GC) into the parent artery is the first step in endovascular treatment. However, in cases where the access route is tortuous, the GC often cannot be appropriately positioned in the target parent artery, therefore, the subsequent procedure becomes difficult. We used the Newton-shaped, 8 French (Fr) Neuro-EBU catheter (SILUX Co., Ltd., Kawaguchi City, Japan) in a total of 21 cases, among which it was successfully used in 19 cases, as a special GC in 14 cases, and for wire exchange in five cases. The 8 Fr Neuro-EBU is a Newton-shaped catheter that is rigid over its entire length except near the tip. In recent years, although there have been presentations of cases treated with Neuro-EBU catheters at academic conferences and seminars in Japan, there are only two published reports describing its use. We report on the usefulness and basic technique of usage of the 8 Fr Neuro-EBU catheter in 21 cases. To the best of our knowledge, there are no previous reports detailing the technical use of the Newton-shaped vascular catheters in the field of neurointervention.

## Materials and methods

Case presentations

Of a total of 424 cases of endovascular treatment performed by the author as an operator at this facility in six years and 11 months from May 1, 2015, to March 31, 2022, 21 cases (4.9%), including four males and 17 females, ranging in age from 64 to 95 years (mean age 78.7 years), involved use of an 8 Fr Neuro-EBU catheter (Table 1). Of the 21 patients in whom the Neuro-EBU was used, 19 cases were successfully treated using the Neuro-EBU. Among these cases, it was used as a GC in 14 cases and as a wire replacement catheter in five cases.

The procedure was unsuccessful in one case in which it was used both as a GC and for wire exchange due to unstable placement of the 8 Fr Neuro-EBU in a bovine aortic arch variant, and in one case in which the catheter broke due to severe tortuosity of the aorta. The treatments performed were coil embolization of an aneurysm in nine cases (one of which was a stent-assisted technique), in all of which the Neuro-EBU was used as a GC, carotid artery stenting in two cases (in both of which it was used as a GC), tumor embolization in two cases (in both of which it was used as a GC), mechanical thrombectomy in six cases (in two of which it was used as a GC), stenting for vertebral artery origin stenosis in one case (used as a GC), and percutaneous transluminal angioplasty (PTA) for basilar artery stenosis in one case (for wire exchange).

The target vessel for the Neuro-EBU was the innominate artery in six cases, the right common carotid artery (CCA) in one case, the right subclavian artery in one case, the left CCA in six cases, the left subclavian artery in five cases, and bovine aortic arch in one case. There was no difficulty in operating the Neuro-EBU in the aortic arch in most of the cases, except in cases 4 and 15. In 14 of the 19 cases in which the 8 Fr Neuro-EBU was used as a GC, stable engagement was possible proximal to the target vessel. Subsequently, a DD6 6 Fr Guide Catheter (Cerulean DD6, Medikit Co., Ltd., Tokyo, Japan) was used as an intermediate distal access catheter (iDAC) for very tortuous blood vessels in seven cases, a 6 Fr Fubuki catheter (Asahi Fubuki 6 Fr, Asahi Intecc Co., Ltd., Aichi, Japan) was used in one case, a 4.2 Fr Fubuki catheter (Asahi Fubuki) was used in one case, a 4 Fr Cerulean inner catheter (Cerulean, Medikit Co., Ltd.) was used in one case and an AXS Catalyst 6 distal access catheter (Stryker Neurovascular, Fremont, CA) was used in one case. It was difficult to introduce the GC or balloon guiding catheter (BGC) by the usual method in three cases of mechanical thrombectomy (cases 9, 10, and 14), one case of PTA for basilar artery stenosis (case 13), and in one case of coil embolization for a ruptured middle cerebral artery aneurysm (case 19). Subsequently, using the Neuro-EBU as a base platform, a stiffer 300 cm long wire was advanced, and the BGC or GC was guided around this by the wire exchange method for subsequent treatment.

In one case with a bovine aortic arch (Case 4) that required mechanical thrombectomy for left middle cerebral artery occlusion, although the Neuro-EBU was initially intended for wire exchange, the wires could not be advanced. Instead, a microcatheter was advanced into the middle cerebral artery from the Neuro-EBU, which was unstably placed at the origin of the bovine aortic arch. In this situation where the intermediate catheter could not be advanced, the microcatheter alone was barely introduced over the lesion and the stent retriever was deployed beyond the occlusion site of the artery. However, since the stent retriever and Neuro-EBU slipped down, the transbrachial approach was instead adopted. In another case that required mechanical thrombectomy for right middle cerebral artery (M2) occlusion (Case 15), we abandoned the procedure due to the breakage of the Neuro-EBU catheter.

**Table 1:** Summary of endovascular treatments performed using an 8 Fr Neuro-EBU catheter Fr: French, F: female, M: male, Rt.: right, Lt.: left, IC-PC: internal carotid artery-posterior communicating artery, CCA: common carotid artery, GC: guide catheter, PCA: posterior cerebral artery, MCA: middle cerebral artery, M1D: M1 distal portion of the middle cerebral artery, VA: vertebral artery, BGC: balloon guiding catheter, PTA: percutaneous transluminal angioplasty, STR: straight

Case No.	Age/Sex	Diagnosis	Treatment	Initial guide catheter used	Target vessel for 8 Fr Neuro-EBU placement	Purpose of using the EBU	Intermediate catheter used (in case of EBU use as a GC)	Microcatheter or stent used (in case of EBU use as a GC)	Outcome of the procedure
1	78/F	Rt. IC-PC unruptured aneurysm	Coiling	EBU used as scheduled	Rt. CCA	As a GC	4.2 Fr Fubuki (latter part of the treatment)	SL10, Echelon10 45º (latter part of the treatment; Marathon)	Completion
2	76/F	Basilar tip unruptured aneurysm	Coiling	EBU used as scheduled	Lt. subclavian artery	As a GC	4 Fr Cerulean	Echelon 10 45º	Completion
3	75/F	Meningioma (rt. middle cranial fossa)	Embolization for meningioma	EBU used as scheduled	Innominate artery	As a GC	Cerulean DD6	Excelsior 1018 STR	Completion
4	66/M	Lt. M1D occlusion	Mechanical thrombectomy	BGC (9 Fr Optimo)	Bovine artery	For wire exchange/as a GC	-	Transit STR	Attempted (failure)
5	64/F	Basilar tip ruptured aneurysm	Coiling	7 Fr GC	Rt. subclavian artery	As a GC	Cerulean DD6	SL10 STR	Completion
6	71/F	Meningioma (lt. cerebral convexity)	Embolization for meningioma	6 Fr GC	Lt. CCA	As a GC	Cerulean DD6	Excelsior 1018 STR	Completion
7	86/F	Lt. PCA dissection (ruptured)	Coiling	8 Fr GC	Lt. subclavian artery	As a GC	Cerulean DD6	Echelon 10 STR	Completion
8	78/F	Unruptured aneurysm (basilar tip)	Coiling	EBU used as scheduled	Lt. subclavian artery	As a GC	Cerulean DD6	Headway 17 STR, Restar 45º (157 cm)	Completion
9	95/F	Lt. M1D occlusion	Mechanical thrombectomy	9 Fr BGC	Lt. CCA	For wire exchange (wire used: 0.035 inch, stiff, 300 cm)	-	-	Completion
10	79/F	Rt. IC (C2) occlusion	Mechanical thrombectomy	9 Fr BGC	Innominate artery	For wire exchange (wire used: 0.035 inch, stiff, 300 cm)	-	-	Completion
11	72/M	Lt. VA origin stenosis (symptomatic)	VA stenting	7 Fr GC	Lt. subclavian artery	As a GC	-	Express vascular SD 5 mm × 15 mm	Completion
12	79/F	Basilar artery aneurysm	Coiling	7 Fr GC	Lt. subclavian artery	As a GC	Cerulean DD6	Phenom 90, Neuroform Atlas 4.5 mm × 30 mm	Completion
13	72/M	Basilar artery stenosis	PTA	EBU used as scheduled	Innominate artery	For wire exchange (wire used: 0.035 inch, extra stiff, 300 cm)	-	Unryu 2.5 mm × 10 mm	Completion
14	85/F	Rt. MCA (M2) occlusion	Mechanical thrombectomy	9 Fr BGC	Innominate artery	For wire exchange (wire used: 0.035 inch, extra stiff, 300 cm)	-	-	Completion
15	91/F	Rt. MCA (M2) occlusion	Mechanical thrombectomy	9 Fr BGC	-	-	-	-	Attempted (failure): breakage of Neuro-EBU
16	72/F	Rt. VA unruptured aneurysm	Coiling	7 Fr GC	Innominate artery	As a GC	Cerulean DD6	SL10 STR (steam shaped)	Completion
17	72/F	Lt. carotid artery stenosis	Carotid artery stenting	9 Fr BGC	Lt. CCA	As a GC	-	Wallstent 8 mm × 21 mm	Completion
18	94/F	Lt. MCA (M1D) occlusion	Mechanical thrombectomy	9 Fr BGC	Lt. CCA	As a GC	Catalyst 6	TREVO TRAK 21 162 cm, Trevo NXT 3 mm × 32 mm	Completion
19	88/F	Rt. MCA ruptured aneurysm	Coiling	8 Fr GC	Innominate artery	For wire exchange (wire used: 0.035 inch, stiff, 300 cm)	-	-	Completion
20	71/M	Lt. CCA stenosis (symptomatic)	Carotid artery stenting	8 Fr BGC	Lt. CCA	As a GC	-	CASPER 10 mm × 30 mm	Completion
21	89/F	Lt. IC-PC ruptured aneurysm	Coiling	8 Fr GC	Lt. CCA	As a GC	6 Fr Fubuki	Phenom 17	Completion

## Results

Case 1

A 78-year-old woman with a history of hypertension came to our hospital for evaluation of headache. An 8 mm posterior communicating artery aneurysm was found, and coil embolization was performed. Preoperative cerebral angiography revealed that it would be difficult to introduce the GC because the right common carotid artery was bent like a hairpin immediately after its origin from the innominate artery.

**Figure 1:**
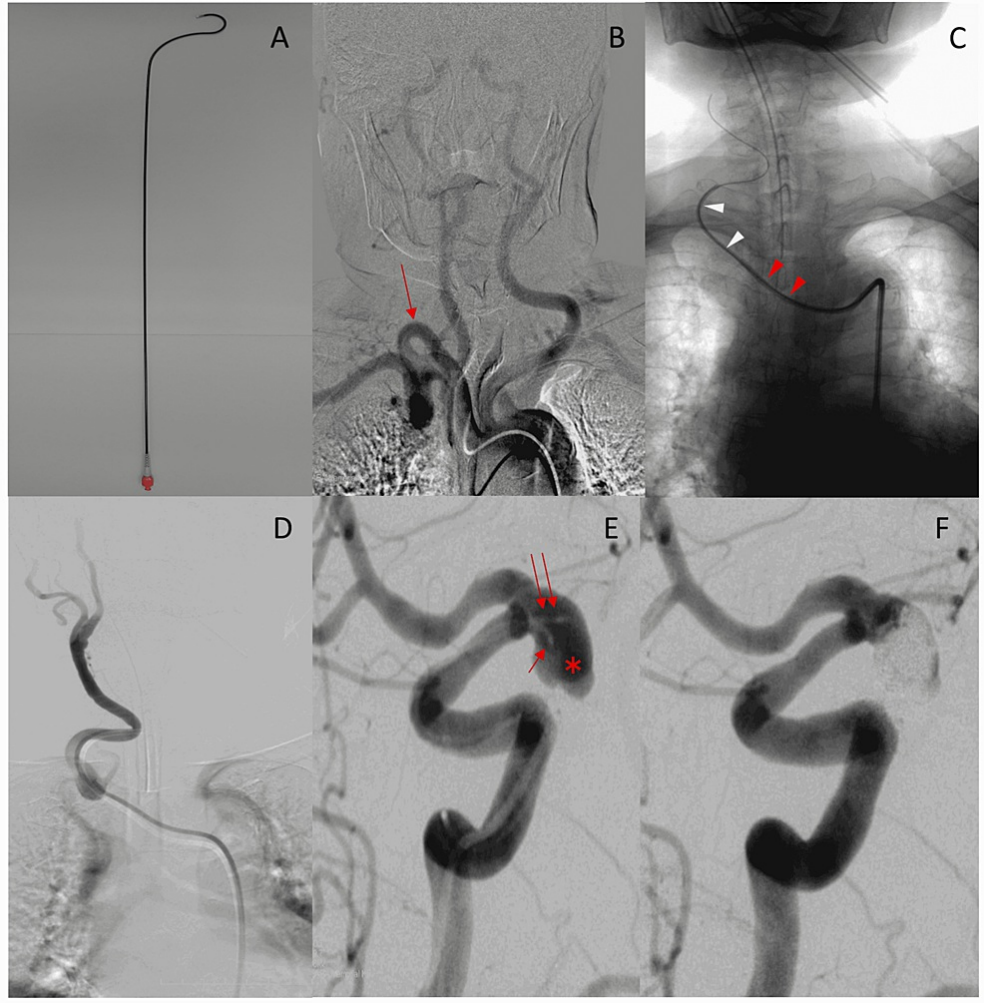
(Case 1) A: Photograph of the 8 Fr Neuro-EBU catheter. The tip of this catheter has a Newton shape, and the total length of the catheter is 83 cm. B: The target vessel, the right common carotid artery (CCA) (arrow), was very tortuous right from its origin. C: An 8 Fr Neuro-EBU (red arrowhead) was introduced into the right CCA through the 6 Fr JB2 type catheter (white arrowhead) with a 0.038-inch guidewire. D: The 8 Fr Neuro-EBU was placed stably into the CCA. E: Double microcatheters (arrow and double arrow) were placed just inside the aneurysm (asterisk: aneurysm). F: The aneurysm was nearly occluded using platinum coils.

First, the 8 Fr Neuro-EBU was placed in the innominate artery via the 6 Fr inner catheter and a 0.035-inch guidewire (GW) was inserted via the right femoral artery. Then, the 0.035-inch GW was changed to a 0.038-inch GW and advanced to the external carotid artery, and the inner catheter was advanced as far as possible using it as a guide (Figure 1C). After slowly advancing and placing the 8 Fr Neuro-EBU in the CCA (Figure 1D), coil embolization was performed using the double catheter technique, constituting the first half of the treatment (Figure 1E). Although a kickback could frequently be felt from the microcatheters that were placed just on the neck side of the aneurysm at the end of treatment, the lack of length made it difficult to operate the catheter. Therefore, we pulled out both the microcatheters and instead used a 4 Fr Cerulean inner GC (Cerulean, Medikit Co., Ltd.) as an intermediate catheter, and inserted a more flexible, longer (165 cm), softer microcatheter (Marathon Flow direct microcatheter, Covidien, Irvine, CA) deeper into the coil ball and completed coil embolization with ED coils (Kaneka Medix, Osaka, Japan) (Figure 1F).

Case 3

**Figure 2:**
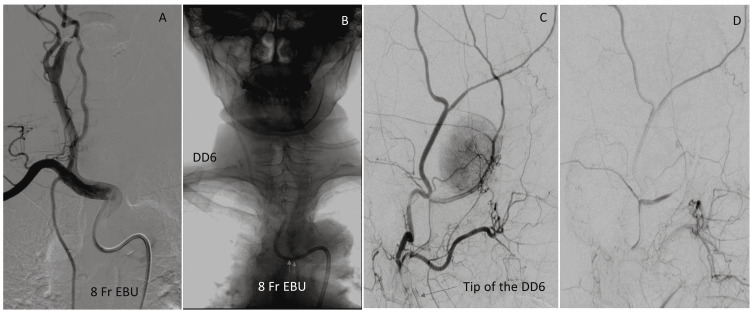
(Case 3) A: Angiogram revealing a type III aortic arch. An 8 Fr Neuro-EBU was placed in the innominate artery. B: A DD6 catheter (arrow) was introduced into the right external carotid artery via the Neuro-EBU (double arrows). C: External carotid angiogram via the DD6 (arrow) showing staining of the meningioma. D: Embolization of the feeding artery of the meningioma with embolic particles and platinum coils was successfully performed, which resulted in the disappearance of staining of the meningioma.

A 75-year-old woman underwent embolization for a 3 cm right middle cranial fossa meningioma. The pattern of the aortic arch was a type III arch, and it was impossible to insert several GWs and inner catheters into the right CCA even with the use of various catheters and GWs. Therefore, an 8 Fr Neuro-EBU was placed in the innominate artery as the GC, and an intermediate catheter (6 Fr Cerulean DD6 GC, Medikit Co., Ltd.) was advanced into the external carotid artery (Figure 2B-2C). The middle meningeal artery and a smaller branch of the internal maxillary artery were successfully embolized (Figure 2D) using an Excelsior 1018 microcatheter (Stryker Neurovascular) and another microcatheter (EchelonTM Straight microcatheter, Covidien), with a thorough dilution of the embolic particles (Embosphere, 300-500 μm, Nippon Kayaku Co., Ltd., Tokyo, Japan) and platinum coils.

Case 8

**Figure 3:**
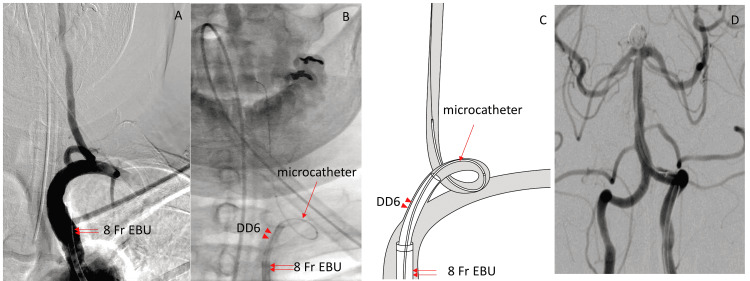
(Case 8) A: Left subclavian angiogram revealed tortuosity of the origin of the vertebral artery. An 8 Fr Neuro-EBU (double arrows) was placed into the left subclavian artery. B: A steam-shaped DD6 catheter (arrowheads) was inserted via the 8 Fr Neuro-EBU (double arrows), and was placed at the origin of the left vertebral artery (VA). A microcatheter (arrow) was advanced distally into the left VA. C: Schema of Figure 3B. D: Two microcatheters (150 cm and 157 cm) were navigated to the basilar tip aneurysm, and the aneurysm was successfully obliterated.

A 78-year-old woman with a history of hypertension was diagnosed with a basilar tip aneurysm during a medical check-up. Since she was extremely anxious about the aneurysm, endovascular treatment was proposed. Coil embolization was performed for the unruptured basilar tip aneurysm. Since severe tortuosity of the origin of the right vertebral artery was confirmed by prior angiography, the approach was made from the left vertebral artery, and it was decided to inject the contrast medium for imaging via the right vertebral artery. However, since there was significant flexion of the origin of the left vertebral artery, the Neuro-EBU was placed in the left subclavian artery and a steam-shaped 6 Fr Cerulean DD6 GC (Medikit Co., Ltd.) was placed at the beginning of the vertebral artery (Figure 3B-3C) through the Neuro-EBU. Since the aneurysm had a wide neck, the double catheter technique was adopted. First, the Headway 17 straight microcatheter (150 cm MicroVention/Terumo, Tustin, CA) was placed in the neck of the aneurysm up to the desired length. However, since the length was a little short, a longer microcatheter (157 cm Restar pre-shaped 45° angled microcatheter, Medico’s Hirata Inc., Osaka, Japan) was placed slightly deeper via the neck of the aneurysm, and coil embolization of the aneurysm was successfully performed.

Case 15

A 94-year-old woman with a history of arrhythmia required mechanical thrombectomy for left middle cerebral artery occlusion (distal M1 occlusion). Due to acute angulation of the left CCA, a JB2 catheter and Simmons type catheter were used as inner catheters through a 9 Fr GC, along with various wires, but the inner catheter could not be advanced. An 8 Fr Neuro-EBU was used as a GC. Using the Neuro-EBU as a platform, mechanical thrombectomy was successfully performed using an aspiration catheter (Catalyst 6 distal access catheter, Stryker Neurovascular) as the intermediate catheter, along with a stent retriever (Trevo NXT ProVue Retriever (3×32) Stryker Neurovascular) that was deployed through a longer 162 cm microcatheter (Trevo Trak 21 microcatheter, Stryker Neurovascular), and treatment was successfully performed.

**Figure 4:**
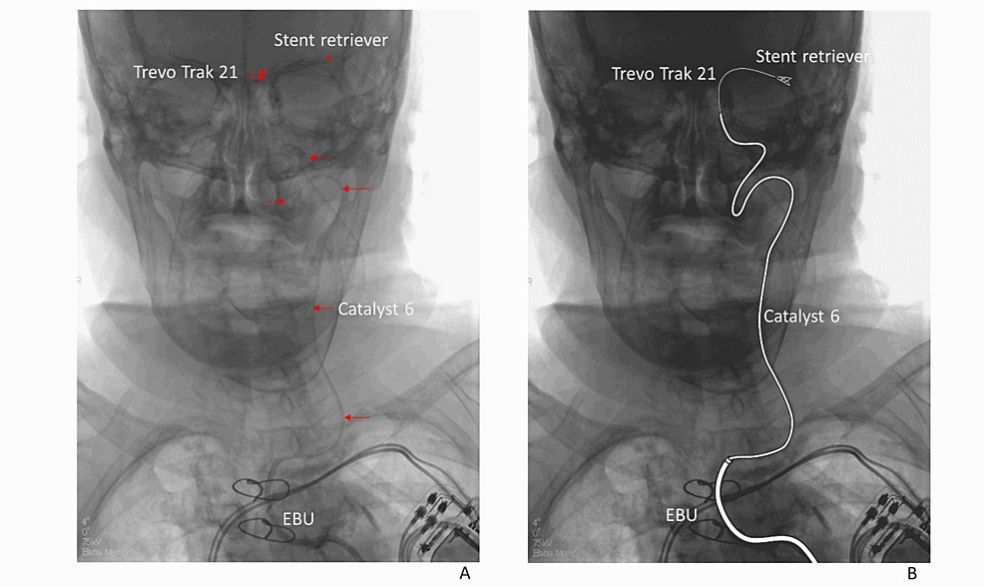
(Case 15) A: The 8 Fr Neuro-EBU was used as a platform and mechanical thrombectomy was successfully performed using an intermediate catheter (Catalyst 6) and Trevo Trak 21 stent retriever (Trevo NTX) through the microcatheter (Trevo Trak 21). Arrows: Catalyst 6, double arrows: microcatheter, arrowhead: stent retriever. B: Schema of Figure 4A.

Case 18

**Figure 5:**
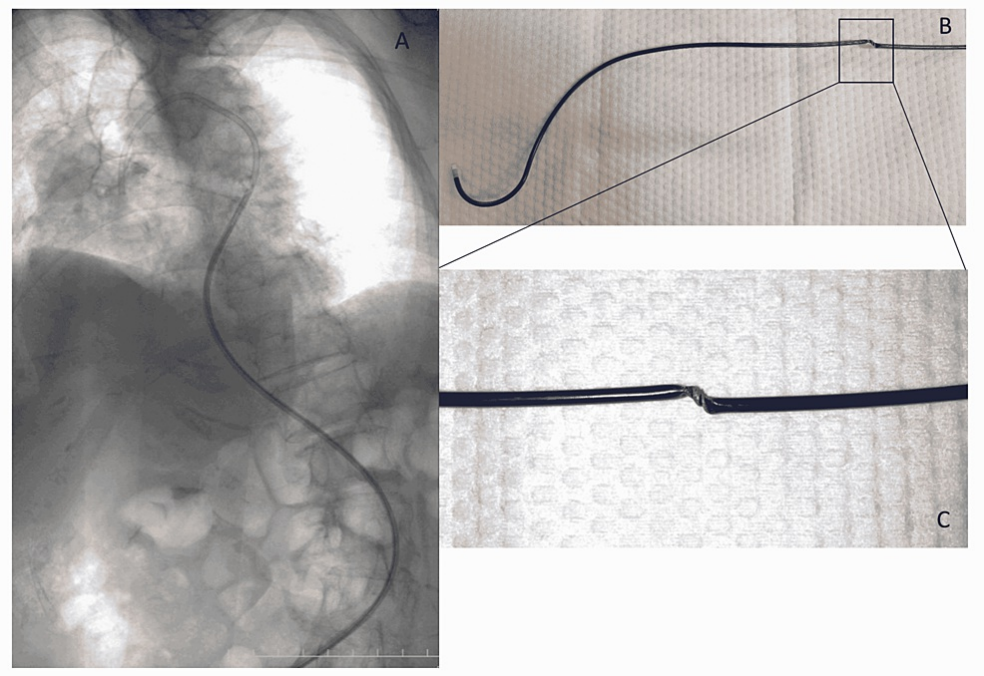
(Case 18) A: Angiogram showing severe tortuosity of the thoracic and abdominal aorta. The Neuro-EBU was manipulated while applying rotation to the ascending or arch aorta. However, due to severe tortuosity of the aorta, the rotational force was not transmitted and the tip did not point in the intended direction. Hence, the use of the 8 Fr Neuro-EBU was abandoned. B: When the 8 Fr Neuro-EBU was completely withdrawn from the body, twisting damage was seen at a site one-third the length of the catheter from the tip. C: Enlarged photo of the damaged point.

A 91-year-old female with impaired consciousness and left hemiplegia underwent mechanical thrombectomy for occlusion of the inferior trunk of the right middle cerebral artery. Severe tortuosity from the femoral artery to the thoracoabdominal aorta and a type 3 aortic arch made it difficult to manipulate the BGC into the right CCA despite the use of several types of GWs and inner catheters. However, since the placement of the GC more distally in the carotid artery was essential, it was decided to advance the BGC to the internal carotid artery using the wire exchange method via the Neuro-EBU. To invert the tip of the catheter and place it in the innominate artery, the Neuro-EBU was manipulated while applying rotation to the ascending or arch aorta. However, due to severe tortuosity of the aorta, the rotational force was not transmitted and the tip did not point in the intended direction. The same operation was repeated many times, but it was still difficult to manipulate the catheter. Thus, the 8 Fr Neuro-EBU was removed. Examination of the catheter demonstrated the presence of twisting damage close to the tip of the 8 Fr catheter, at a distance of one-third the length of the catheter from the tip (Figure 5B-5C). Since this case was a very elderly patient with right middle cerebral artery (M2) occlusion, and the natural history of her disease was relatively good, the mechanical thrombectomy procedure was abandoned due to the risks associated with continuing endovascular therapy.

## Discussion

Neuroendovascular treatments have undergone remarkable advances in recent years and continue to do so [1-4]. The first step in endovascular treatment is delivering and achieving stable placement of the GC in the target artery [5-7]. However, since it is sometimes difficult to obtain sufficient stabilization of the catheter because of the severe tortuosity of the approach route [8], endovascular treatment cannot always be completed. The authors previously reported the usefulness of the Amplatz GooseNeck snare (ev3, Plymouth, MN, USA) for advancing and folding the GC [9]. In the current case series, the Neuro-EBU catheter used in the 21 cases (Table 1), which is manufactured by Silux Co., Ltd. and sold by Hanako Medical Co., Ltd. (Saitama City, Japan), had an outer diameter of 8 Fr, an effective length of 83 cm, and an inner diameter of 0.090 inches (Figure 1A). This catheter has a Newton shape with a stainless blade processed with polyamide on the outer layer and polytetrafluoroethylene on the inner layer. The flexible part at the tip has a length of 5 mm, with the distal-most 1 mm being soft, while the remaining part of the catheter is very rigid over its entire length. In recent years, although there have been some presentations at Brush-up Seminars of Neuroendovascular Therapy (BSNET), the Japanese Society for Neuroendovascular Treatment (JNET), The Japan Neurosurgical Society, and other interventional radiology seminars in Japan, in terms of published papers, only two reports related to use of the Neuro-EBU have been published [10] [1]. In one of them, Ishii referred to the Neuro-EBU as “the ultimate weapon in times of inaccessibility” of the target vessel [10].

The basic method of using the catheter is to engage the Neuro-EBU with respect to the three branches of the aorta (innominate artery, left CCA, and left subclavian artery) via the aorta and to use the Neuro-EBU as a GC or to use it for GW exchange. In the current series, we used the Neuro-EBU as a GC in 14 of 21 cases, and as part of the wire exchange method to advance a standard GC in five of the remaining cases. In our series, the Neuro-EBU could be stably placed into the intended target vessels from the aortic arch in all but two of the cases (Cases 4 and 15) (Table 1). When guiding the Neuro-EBU into the intended target vessel, it is basically guided from the femoral artery to the aortic arch via a 6 Fr JB2 inner catheter and 0.035-inch GW. Thereafter, the tip of the Neuro-EBU itself is directed toward the head while slowly rotating it at the level of the ascending aorta and aortic arch. The next step is to hook the tip of the catheter while carefully pushing and pulling it to the target branch, as with a Simmons-type catheter. If it is not possible to engage the Neuro-EBU into the target vessel in this way, it is advisable to hook the inner catheter together with the 0.035-inch GW into the target vessel in advance and then insert the Neuro-EBU along with them. Ishii stated that the Neuro-EBU cannot be guided beyond the aorta because the Newton-shaped 8 Fr Neuro-EBU is very rigid [10]. However, in our series, in both Case 1 (Figure 1) and Case 5, after its engagement with the target vessel using the basic operation method of the Neuro-EBU in the aorta, the Neuro-EBU could be further advanced to the right CCA and the right subclavian artery, respectively, as far as possible, along with the JB2 catheter and the GW placed further distally in advance.

The next step after placing the Neuro-EBU as a GC, which helps to create a stronger foundation, is the use of the iDAC. We used an iDAC in 11 of the 14 cases in which the Neuro-EBU was used as a GC (Table 1). Here, as was also seen with case 1, another point to keep in mind besides using an iDAC when using the Neuro-EBU as a GC is whether the microcatheter can be sufficiently distally advanced. In case 8 (Figure 3), when performing the double catheter technique, a 150 cm Headway 17 microcatheter (Straight microcatheter, MicroVention/Terumo, Tustin, CA, USA) barely reached the neck of the aneurysm. Therefore, another longer microcatheter (Restar 157 cm, Medico’s Hirata, Osaka, Japan) was inserted slightly deeper into the aneurysm, and coil embolization was successfully performed by entwining the coils from both microcatheters. In cases with severe tortuosity, it is difficult to advance the iDAC distally. Currently, a two-marker 150 cm microcatheter is commonly used, although the more distally placed iDAC and the length of the microcatheter determine the accessibility of the aneurysm, especially when using the Neuro-EBU as a GC for coil embolization of aneurysms, as in Cases 1 and 8 (Figure 1, Figure 3).

In cases with severe tortuosity, in which the intermediate catheter cannot be advanced distally enough, treatable aneurysms are limited to those that are more proximally located. In one mechanical thrombectomy case (Case 18) in this series, we successfully used the Neuro-EBU as a GC along with an aspiration catheter (Catalyst 6) as the iDAC, which could be guided further distally, and a 162 cm long Trevo Trak with a microcatheter (Figure 4D-4E). It is expected that the combination of a flexible iDAC, a wide variety of which are currently available, and a long microcatheter will increase the number of cases with severe tortuosity of the target blood vessel that can be treated by mechanical thrombectomy. If a longer catheter is not available, another option is to place a connector attached to the catheter with a shorter connector right from the outset of the treatment.

In all five cases in which the Neuro-EBU was used for wire exchange, the procedure was successfully performed. In Cases 9, 10, 13, and 19, a 300 cm 0.035-inch stiff wire was used due to the Neuro-EBU providing a firm platform, and, in Case 14, using an Amplatz extra stiff GW (THSF-35-300-AES GW, 0.35 inch, 300 cm, Cook Medical, Bloomington, IN, USA), and following the advancement of these wires, the EBU was removed and a BGC with a 6 Fr inner catheter was advanced along the wire, with subsequent successful endovascular treatment.

The Neuro-EBU was unsuccessful in two of our cases. In Case 4, which had a bovine-type aortic arch, we tried using a Simmons-type inner catheter and various wires, but it was difficult to advance the BGC. Therefore, the Neuro-EBU was used, although, due to the bovine arch, the tip of the Neuro-EBU did not point in the direction of the left CCA. Finally, since the wires could not be advanced despite attempts with various wires, the wire replacement method was abandoned. While the assistant manually fixed the Neuro-EBU while continuously applying torque in the direction of the left CCA, the microcatheter was directly advanced to the occluded lesion in the middle cerebral artery and mechanical thrombectomy with the stent retriever was attempted once, although the procedure was unsuccessful because the intermediate catheter could not be advanced during deployment of the stent retriever, and the stent itself and the Neuro-EBU slipped off. In this case, we eventually changed to the transbrachial approach [11] [2], since despite the rigidity of the Neuro-EBU and how well it serves as a platform for the passage of other catheters, it is difficult to continue treatment in cases in which its placement is unstable. In such cases, alternative methods, such as the transbrachial approach, or introducing and snaring the BGC or Neuro-EBU itself with a Goose Neck snare [9], and direct carotid artery puncture should be considered [12] [13].

In case 15, since manipulation of the 8 Fr Neuro-EBU in the aortic arch did not adequately transmit the rotation force to the tip of the catheter, the 8 Fr Neuro-EBU was abandoned. On withdrawal of the catheter, it was seen to have undergone twisting damage (Fig. 5B, C). Since this patient was elderly and had severe arterial tortuosity from the abdominal aorta to the aortic arch, the Neuro-EBU could not be manipulated. Our experience suggests that even though the Neuro-EBU is rigid over its entire length, in cases where the rotation force is not transmitted to the tip, it could result in damage to the Neuro-EBU itself, leading to vascular damage or cholesterol embolism due to excessive rotation or the push-pull maneuver. Hence, the procedure should be abandoned and an alternative technique adopted in such cases.This paper has the limitation of reporting on a small sample size and should be verified with further use in the future.

## Conclusions

The 8 Fr Neuro-EBU is rigid over its entire length and has an excellent backup function as a firm platform for the passage of other catheters. In some cases where the stable placement of a standard GC is difficult, treatment can be performed by placing the Neuro-EBU proximal to the target vessel as a basic GC in combination with an intermediate catheter. It might also be useful when advancing the exchange wire. Although not frequently used when performing endovascular treatments, the Newton-shaped Neuro-EBU catheter can be a trump card in cases where access routes are difficult.
